# Challenges in undertaking non-linear Mendelian randomization

**DOI:** 10.1002/oby.23927

**Published:** 2023-10-16

**Authors:** Kaitlin H. Wade, Fergus W. Hamilton, David Carslake, Naveed Sattar, George Davey Smith, Nicholas J Timpson

**Affiliations:** 1 Medical Research Council (MRC) Integrative Epidemiology Unit, University of Bristol, Bristol, UK; 2 Population Health Sciences, Bristol Medical School, Faculty of Health Sciences, University of Bristol, Bristol, UK; 3 Infection Science, North Bristol NHS Trust; 4 Institute of Cardiovascular and Medical Sciences, British Heart Foundation Glasgow Cardiovascular Research Centre, University of Glasgow, Glasgow, UK

## Abstract

Mendelian randomization (MR) is a widely used method exploiting the unique properties of germline genetic variation to strengthen causal inference in relationships between exposures and outcomes. Non-linear MR allows estimation of the shape of these relationships. In a previous paper, we applied linear and non-linear MR to estimate the effect of body mass index (BMI) on mortality in UK Biobank, providing evidence for a J-shaped association. However, it is now clear that there are problems with widely used non-linear MR methods, drawing attention to the likely erroneous nature of the conclusions regarding the shapes of several explored relationships. Here, we explore the utility and likely biases of these non-linear MR methods with the use of a negative control design. Whilst there remains good evidence for a causal effect of higher BMI increasing the risk of mortality, the pattern of this association across different levels of BMI requires further characterisation.

Mendelian randomization (MR) is a widely used method exploiting the unique properties of germline genetic variation to strengthen causal inference in observational epidemiological studies^1^. There have been many developments in MR methodology, which expand the scope of what can be estimated and provide many sensitivity analyses that facilitate robust inference. One of these developments is non-linear MR, which apparently allows estimation of the shape of the relationship between an exposure and outcome^2^. One observational epidemiological association that is often reported as non-linear is that between body mass index (BMI) and mortality, which is markedly J-shaped. In a paper published in *Obesity*, we applied linear and non-linear MR to estimate the effect of BMI on mortality in UK Biobank^3^. Using a linear MR model, there was evidence for a causal effect of BMI on the risk of both all-cause mortality and mortality from specific causes including cardiovascular disease (CVD). For example, the hazard increased by approximately 3% (hazard ratio (HR): 1.03; 95% CI: 0.99-1.07) for all-cause mortality and by approximately 10% (HR: 1.10; 95% CI: 1.01-1.19) for mortality from CVD with every kg/m^2^ higher BMI.

In the same work, the then relatively new non-linear MR approach^2^ was applied to characterise the shape of the associations. This method involves calculating the residuals from regression of measured BMI on the genetic instrument and undertaking MR analyses in strata defined by the “instrument-free”, residualised BMI. This avoids the collider bias that would be generated by simply stratifying on measured BMI^2^. In these analyses, there was weak evidence for a J-shaped association between BMI and mortality. Indeed, a later analysis using both UK Biobank and the Nord-Trondelag Health (HUNT) Study found similar results using the same method, although these results were likely complicated by additional stratification by smoking status^4^, which itself could cause collider bias since BMI influences smoking behaviour^5^.

It is now evident that there are serious problems with this widely used non-linear MR method^6, 7^. In an application to vitamin D and mortality^8^, impossible findings were generated^7^. The authors of the residualisation method fully accept that the strikingly non-linear results generated are spurious^6^ and the journal has issued an “expression of concern” regarding the paper^9^.

It appears highly likely that errors will be generated in other applications of the non-linear MR approach, which has led us to draw attention to the likely erroneous nature of the conclusions regarding the shape of the relationship between BMI and mortality in our paper. We note, however, that our conclusions regarding the linear MR finding remain unchanged. To uncover potential problems with the non-linear MR approach, we have applied negative control methodology, which uses a factor in the analyses as the outcome that cannot possibly be altered by the exposure in question^10^. It is clear that BMI cannot influence assigned sex; therefore, we applied the same non-linear MR analytical strategy that we had used in our paper in a comparable sample of UK Biobank, but replaced mortality with assigned sex as the outcome (Figure 1). The figure shows that the method produces the nonsensical finding that lower BMI makes participants less likely to be male, whereas high BMI renders them more likely to be male. Such bias may be induced by issues such as non-constant genetic effects across the range of BMI, or to differential selection bias within the strata of residual BMI, amongst other possibilities.

In their letter acknowledging that their residualisation method was flawed, the authors advanced a supposedly more robust “doubly-ranked” non-linear MR approach^6^. Our figure shows that this also fails a sanity check using assigned sex as a negative control outcome^10^.

When applying conventional MR methodology (i.e., assuming linearity in the relationships between the genetic instrument(s) and the exposure and between the exposure and outcome), the relationship between BMI and assigned sex was far weaker (odds ratio for being male with each kg/m^2^ higher BMI: 1.02; 95% CI: 1.01, 1.03). This is in clear contrast to the magnitude of estimates provided within BMI strata (Table 1) and the coincident heterogeneity in estimates across strata of BMI in the non-linear MR analyses (where heterogeneity p-values across strata for both residual and ranked methods were essentially zero). Nevertheless, these results continue to highlight the requirement for careful interpretation of imperfect MR estimates in light of complexity in polygenic instrumentation measurement and sampling frames.

Due to ongoing concern about the reliability of the non-linear MR methodology on which we focused here (flagged by the extent of likely biases generated) and in the absence of a definitive answer as to the magnitude of impact biases of this nature elsewhere, we feel it necessary to state that the non-linear MR findings included in our original paper, published in *Obesity*, may well be unreliable. Unfortunately, we think the same concern applies to the findings in the >70 other papers that have been published using this method.

The application and interpretation of advanced statistical methods often requires many assumptions to be met. Whilst there remains good evidence for a causal effect of higher BMI increasing the risk of mortality, the pattern of this association across different levels of BMI requires further characterisation. Currently, the exact source of error generated by both the original and the newly proposed non-linear MR methods is unclear and we suggest there should be a moratorium on further applications of them until there is better evidence regarding reliability. This example provides yet more evidence as to why triangulation of evidence across multiple epidemiological study designs with orthogonal biases is necessary for reliable interpretation. In this case, the use of a negative control outcome casts the findings into doubt, as it would have done for the impossible results that were published regarding vitamin D and mortality.

## Figures and Tables

**Figure:**
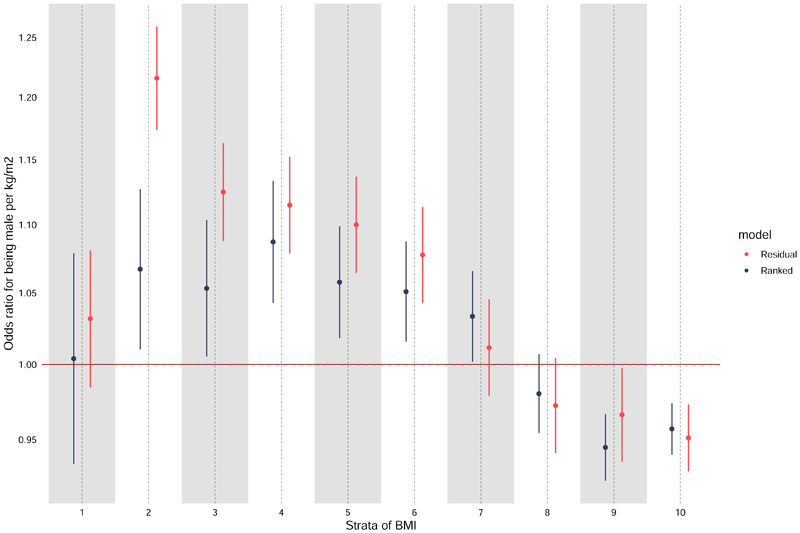


**Table 1:** Local averaged causal effect (LACE) estimates using Mendelian randomization (MR) from the negative control study showing the “causal effect” of BMI on assigned sex within strata of BMI using UK Biobank data comparing the residual and ranked methods

Strata of BMI	Beta	SE	LCI	UCI	P
**Residual**					
1	0.03	0.02	-0.02	0.08	0.19
2	0.20	0.02	0.16	0.23	2.49x10^-27^
3	0.12	0.02	0.08	0.15	5.38x10^-12^
4	0.11	0.02	0.08	0.14	1.09x10^-10^
5	0.10	0.02	0.06	0.13	1.27x10^-08^
6	0.07	0.02	0.04	0.11	8.24x10^-06^
7	0.01	0.02	-0.02	0.04	0.49
8	-0.03	0.02	-0.06	0.004	0.09
9	-0.03	0.02	-0.07	-0.002	0.03
10	-0.05	0.01	-0.07	-0.03	1.77x10^-05^
**Ranked**					
1	0.004	0.04	-0.07	0.08	0.91
2	0.07	0.03	0.01	0.12	0.02
3	0.05	0.02	0.01	0.10	0.03
4	0.08	0.02	0.04	0.13	8.36x10^-05^
5	0.06	0.02	0.02	0.09	0.004
6	0.05	0.02	0.02	0.08	0.004
7	0.03	0.02	0.002	0.06	0.04
8	-0.02	0.01	-0.05	0.01	0.15
9	-0.06	0.01	-0.08	-0.03	1.06x10^-06^
10	-0.04	0.01	-0.06	-0.03	8.51x10^-07^

BMI = body mass index in kg/m^2^; LCI = lower confidence interval; SE = standard error; UCI = upper confidence interval. Betas represent log odds ratios for being male with each kg/m^2^ higher BMI. Here, to avoid possible bias, the analysis was conducted among people where their self-reported sex was consistent with their sex chromosomes (i.e., XY for males and XX for females), where the reference was “female”. After calculating the residual or ranked BMI distribution for the application of each method, BMI strata were defined by splitting the resulting continuous residual or ranked BMI distribution into ten equal quantiles.

## What is already known about this subject? Please remember to also include this between the title page and structured abstract in your paper.

- Mendelian randomization (MR) is a widely used method exploiting the unique properties of germline genetic variation to strengthen causal inference in relationships between exposures and outcomes, where non-linear MR allows estimation of the shape of these relationships.
- There is a J-shaped relationship between body mass index (BMI) and mortality reported in previous observational studies and apparently in non-linear MR analyses.
- It is now clear that there are problems with non-linear MR methods.

## What are the findings in your manuscript? Please remember to also include between the title page and structured abstract in your paper.

- Using a negative control design, we demonstrate bias in analyses utilising two commonly used non-linear MR methods.
- Whilst there remains good evidence for a causal effect of higher BMI increasing the risk of mortality, the pattern of this association across different levels of BMI requires further characterisation.

## How might your results change the direction of research or the focus of clinical practice? Please remember to also include between the title page and structured abstract in your paper.

- Whilst the source of error generated with commonly used non-linear MR methods is currently unclear, we suggest that there should be a pause in publication of such studies until there is better understanding regarding their reliability.
- We provide further support as to why triangulation of evidence across multiple study designs with orthogonal biases is necessary for reliable interpretation.

## References

[1] Richmond RC, Davey Smith G (2022). Mendelian Randomization: Concepts and Scope. Cold Spring Harbor Perspectives in Medicine.

[2] Staley JR, Burgess S (2017). Semiparametric methods for estimation of a nonlinear exposure-outcome relationship using instrumental variables with application to Mendelian randomization. Genet Epidemiol.

[3] Wade KH, Carslake D, Sattar N, Davey Smith G, Timpson NJ (2018). BMI and Mortality in UK Biobank: Revised Estimates Using Mendelian Randomization. Obesity (Silver Spring).

[4] Sun Y-Q, Burgess S, Staley JR, Wood AM, Bell S, Kaptoge SK, Guo Q, Bolton TR, Mason AM, Butterworth AS, Di Angelantonio E (2019). Body mass index and all cause mortality in HUNT and UK Biobank studies: linear and non-linear mendelian randomisation analyses. BMJ.

[5] Carreras-Torres R, Johansson M, Haycock PC, Relton CL, Davey Smith G, Brennan P, Martin RM (2018). Role of obesity in smoking behaviour: Mendelian randomisation study in UK Biobank. BMJ.

[6] Burgess S, Wood AM, Butterworth AS (2023). Mendelian randomisation and vitamin D: the importance of model assumptions – Authors’ reply. The Lancet Diabetes & Endocrinology.

[7] Davey Smith G (2023). Mendelian randomisation and vitamin D: the importance of model assumptions. Lancet Diabetes Endocrinol.

[8] Sofianopoulou E, Kaptoge SK, Afzal S, Jiang T, Gill D, Gundersen TE, Bolton TR, Allara E, Arnold MG, Mason AM, Chung R (2021). Estimating dose-response relationships for vitamin D with coronary heart disease, stroke, and all-cause mortality: observational and Mendelian randomisation analyses. The Lancet Diabetes & Endocrinology.

[9] (2023). Expression of Concern—Estimating dose-response relationships for vitamin D with coronary heart disease, stroke, and all-cause mortality: observational and Mendelian randomisation analyses. The Lancet Diabetes & Endocrinology.

[10] Sanderson E, Richardson TG, Hemani G, Davey Smith G (2021). The use of negative control outcomes in Mendelian randomization to detect potential population stratification. International Journal of Epidemiology.

